# Sport for development sector and climate change adaptation in Zambia—theories of strategic choice

**DOI:** 10.3389/fspor.2026.1756982

**Published:** 2026-07-01

**Authors:** Davies Banda, Derrick Charway, Collins Kaluba

**Affiliations:** 1 Moray House School of Education and Sport, ISPEHS, The University of Edinburgh, Edinburgh, United Kingdom; 2 School of Postgraduate Studies, University of Lusaka, Lusaka, Zambia; 3 Department of Sport and Social Sciences, Norwegian School of Sport Sciences, Oslo, Norway; 4 Department of Primary Education, University of Zambia, Lusaka, Zambia

**Keywords:** climate change, environmental determinism, environmental stewardship, sport-for-development and peace, strategic choice, voluntarism, Zambia

## Abstract

The World Economic Forum Annual Report 2023–2024 warned of the devastating impact that climate change will have on human health by 2025, claiming that 14.5 million deaths by 2050 are likely to occur because of weather-induced catastrophes such as droughts, floods, extreme heat, heatwaves, wildfires, and tropical storms. Regions of the world, whether in the Global North or Global South, are experiencing different, if not multiple, forms of these climate-driven events. In the Global South, home to many sport-for-development non-governmental organisations (SDP NGOs), community actors are responding to the call for environmental stewardship. The El Nino-induced drought in Zambia has destabilised the operation of SDP NGOs as organisations and participants experience water shortages, erratic electricity supply, and limited engagement time with participants. This study purposed to advance environmental research among some of Zambia's SDP sector actors by demonstrating their engagement in national climate adaptation programmes. The study explored how the SDP sector is responding to environmental matters such as the prolonged drought in Southern Africa and its implications on the operations of the SDP sector. Drawing upon John Child and Richard Whittington's theories of strategic choice, the study analysed the structural constraints and agency of SDP NGOs regarding climate education and practical adaptations to external forces. This study utilised a survey questionnaire which was administered among SDP actors and semi-structured interviews with purposively selected SDP actors to highlight organisational responses to climate change impacts. Using a realist lens, this study examined how agency and structure interact through SDP actions. The study reveals that SDP actors have responded through climate adaptation actions in efforts to transform their context amid the harsh local realities of climate change. It further reveals that SDP NGOs’ climate education continue to be constrained by environment and economic determinants.

## Introduction

In the global South, Sport for Development and Peace (SDP) non-governmental organisations (NGOs) have played a significant role in embracing and acting upon global goals such as the United Nation's eight (1) Millennium Development Goals (MDGs) and currently the seventeen Sustainable Development Goals (SDGs) (2). Through this paper, we hope to extend the discussion that was started by Giulianotti and others when they questioned the often-marginal status that is accorded to environmental issues by the SDP sector. The SDP sector is globally recognised as composed of those organisations and actors who use sport as an accelerator for peace and sustainable development for all (3), use of sport as a hook to engage target groups with intentions to delivery sociocultural development outcomes (4, 5), and sport as a vehicle for development and peacebuilding (6), to mention but a few intentional instrumental usages of sport and physical activity to addressing non-sporting outcomes (4).

SDP organisations have a proven track record of aligning their activities to global development issues which have provided impetus for the recognition of the sector within international development (2). The sector's use of sport as a vehicle for broader developmental outcomes encompasses areas such as sexual-health education, youth empowerment, and gender equity (7). However, while visible in such endeavours aligned to the SDGs, the SDP sector's engagement with environmental sustainability and climate change has been notably limited (8). Indeed, mainstream SDP programming has historically side-lined environmental sustainability issues, with climate change rarely integrated into core objectives (8). This oversight is particularly concerning given that the physical environment is a major theme in both the research and practice of international development and features prominently in the SDGs.

Climate change presents an escalating and multifaceted threat to global development, with particularly acute implications for sub-Saharan African nations like Zambia, the location of this study (1). Zambia faces increased average temperatures and erratic rainfall patterns, leading to severe droughts and floods that exacerbate socio-economic decline (9). These climate impacts disproportionately affect vulnerable populations, including women and girls, and pose significant threats to national development, economic systems, livelihoods, and ecosystems (10). The aforementioned vulnerable groups are the primary focus of sport-based interventions conducted by SDP organisations. The imperative for robust climate adaptation strategies is therefore urgent, demanding integrative approaches, which promote active and meaningful participation of all key stakeholders across multisectoral initiatives (11).

SDP NGOs in Zambia have played significant roles in localising global policy agendas at the micro-level for issues such as global health pandemics and gender mainstreaming (11). However, their active engagement at the national level in matters pertaining to environmental sustainability and climate resilience has historically not been prominent in SDP programming (8). This indicates a critical gap where SDP actors seem to operate on the periphery of environmental sustainability and climate resilience planning, despite their proximity to and understanding of local contexts and needs within climate-vulnerable communities. This paper questions that status, the disconnect of the three pillars in what is commonly known as composing sustainable development: environment, economy and society (12). This disconnect is evidenced by the marginal importance accorded to environmental issues within the SDP sector, challenging the sector to reconsider its practices regarding environmental sustainability. While sustainability literature (13) suggests that poverty causes environmental degradation, other studies (14) have also highlighted institutional failures as root causes of environmental degradation. The SDP sector in Zambia emerged out of such institutional failures by government (15) under the one-party political rule which consequently pushed the Zambian population into severe poverty following expanding international debt. SDP NGOs have traditionally concentrated on alleviating the effects of poverty while also striving to address its root causes (7). Nevertheless, the relationship between poverty and environmental degradation, although centred on natural resources, has not been prominently addressed within SDP initiatives, which also aim to alleviate poverty.

This paper is therefore justified by the critical need to explore how SDP actors in Zambia are responding to climate variability and change, particularly within a region where the devastating impacts of climate change on the Zambian economy highlight the urgent need for adaptation. This research will contribute to understanding how SDP organisations have responded to the call to action towards environmental matters whose amplification has come through prolonged droughts and subsequent power outages not only in Zambia but across the southern African region.

The following section provides a contextual overview of climate change and its adverse effects on national development, livelihoods, and ecosystems. This discussion situates SDP organisations within the context of these climate-related vulnerabilities while critically examining both local and global SDP sectors’ limited engagement with environmental concerns. This is followed by a section detailing the methodologies used to collect empirical data, which aim to elucidate shifts in attention toward environmental concerns in response to a severe El Niño-induced drought in Zambia. The results and discussion of insights are then presented, preceding a concluding section that examines the implications of these findings.

### Contextualising climate change in Zambia

Zambia, similar to other sub-Saharan African countries, faces a significant threat from the negative implications of climate change, with projected increase in higher average temperatures and reduced rainfall patterns (1, 9). These climate change effects have resulted in droughts and floods which have exacerbated the decline of socio-economic development gains (16, 17). Zambia faces several challenges towards effective climate change adaptation (18). Such challenges include economic, political, environmental, and social factors which stand in the way of effectively implementing adaptive practices (10, 19). Addressing the mentioned challenges necessitate robust adaptation strategies to ensure resilience against the current and future impacts of a changing climate.

While the impacts of climate change are felt globally, the disproportionate burdens of what some have termed climate of injustices (20) are experienced more by those in the Global South. Countries in the Global South are experiencing these lop-sided burdens due to structural vulnerabilities and constrained adaptive capacities according to a report by the World Meteorological Organisation (WMO) (21). While other commentators (22, 23) contend that, despite bearing the greatest burdens of climate change, these regions of the world contribute minimally to the drivers exacerbating climate related catastrophes.

As a low-middle income country, Zambia is facing the disproportionate burdens of climate change, posing a significant and multifaceted threat to national development. The imperative for robust adaptation strategies is underscored by the nation's vulnerability (19), particularly among its most susceptible populations composed mainly of a youthful population. A critical aspect of effective climate change adaptation in Zambia is the active and meaningful participation of all key stakeholders, including community-based organisations (CBOs) and SDP NGOs.

Similar to other nations, building climate resilience is critical for development which is inclusive and sustainable. In a 2021 policy belief, the Government of Zambia outlined processes for National Adaptation Planning (NAP) which called for an integrated climate change adaptation (CCA) (19). Among the key challenges outlined in the policy brief which related to local SDP practices was building resilience of women, young people and other vulnerable groups in communities. To do this, integration into Disaster Management and Mitigation Unit's (DMMU) coordinated national and sub-national level structures was stressed as vital for engaging with civil society organisations and other key stakeholders (19). However, the role of sport entities remained invisible in policy deliberations until recent developments captured in this study where sport is seen responding to climate related matters. These emerging SDP practices tend to be specific to the 2023–24 El Nino period where 2023 is considered to record the worst catalogue of climate-related disasters in Southern Africa (21).

This paper considers such organisations which have played a significant role in grassroot development as instrumental in fostering learning for other sustainable development initiatives. CBOs have been known to closely work with communities they serve, drawing local knowledge from grassroot actors to instigate behavioural and social change critical for nurturing best practices regarding adaptation. Similar to the role that such SDP NGOs have played in contributing to the fight against global health pandemics such as HIV/AIDS (15, 24, 25), these organisations have a vital role in ensuring adaptation efforts are contextually relevant to addressing the needs of vulnerable communities they normally serve. CBOs are uniquely positioned to facilitate these targeted adaptation actions due to their proximity to and understanding of local contexts and needs (26). However, there is inadequate evidence of such involvement among sport-based CBOs in Zambia.

### SDP and the physical environment

CBOs operating within the SDP operating space have used sport as a vehicle for broader developmental outcomes (27, 28) which include health education, youth empowerment, and gender equity (29, 30). However, despite the widely publicised potential of sport to foster social transformation, scholars caution against uncritical optimism. Scholars in SDP argue that claims of sport's developmental efficacy often lack rigorous empirical validation and risk obscuring deeper structural inequalities (31, 32). This critique is particularly salient in the climate context, where systemic environmental threats demand more than temporary or symbolic interventions. While some CBO actors are beginning to engage in environmental and climate activism, mainstream SDP programming has historically side-lined issues of environmental sustainability, with climate change rarely integrated into core objectives (33).

SDP NGOs in the Global South, have been known to play a significant role as mediators between global policy agendas and local lived realities (34). Highlighting the role of indigenous SDP actors in Zambia, Lindsey and others point out SDP NGOs’ active role in localising of global issues depicted in the SDGs at micro-level (7). However, Moura and Scott (35) argue that while a range of SDGs are given attention through SDP programming, there lacks specific focus to environmental sustainability given the ongoing climate crisis. Giulianotti and others (8) having pointed out this already that:

…the physical environment is a major theme in both the research and practice of international development that has been largely neglected by both SDP stakeholders and scholars. …[an] oversight reflected in the often-marginal, status afforded to environmental issues within programmes, campaigns, and initiatives that take place within the SDP sector, as well as the general paucity of SDP research that focuses on the physical environment.

These authors claim that despite the physical environment's wide recognition through most of the SDGs, there is less attention that has been given to environmental programming among SDP organisations. Notwithstanding the devastating direct impacts of climate change, SDP actors in the Global South have yet to demonstrate structured environmental sustainability planning and programme delivery as seen in other sectors such as tourism (36). Those operating within climate-vulnerable communities, have a role to play in translation of global environmental mandates into local context-specific action. Contextualising to local needs is not a novel approach for SDP actors; rather, it is a process that necessitates incremental adaptations, as these actors have previously demonstrated with other development initiatives such as gender equality and HIV/AIDS (7, 11, 34).

Hence, research by Giulianotti and others challenges the SDP sector to reconsider SDP practices regarding the importance and significance of environmental sustainability. Drawing upon Giddings, Hopwood and O’Brien's work, it seems that the SDP sector have not demonstrated the interconnectedness of the three pillars of sustainable development—environment, society and economy (37). Rather, observations in SDP literature reveal that the sector's gaze has been more on society and the economy with less attention to the environment (8). Giddings and others argue that “the economy [is] dependent on society and the environment while human existence and society are dependent on, and within the environment”. Furthermore, they argue that 'sustainable development needs to be based on principles that would apply to all issues whether they are classified as environmental, social, economic or any mix of the three’.

For the SDP sector, its claim to long-term sustainable development demands an acknowledgement of these interconnections as the environment seems to feature prominently within the sustainable development goals (8). Despite the depiction of sustainable development as a relationship between three interconnected equal sized rings (37), the SDP sector seems to have focused more on sociocultural aspects surrounding equity and participation. Historically, the SDP sector has given priority to socio-economic issues, gender inequalities and poverty of vulnerable populations despite the visible and invisible environmental causes to development concerns. SDP scholars who have advocated for deep critical outlook call for programming that acknowledges and makes attempts to tackle the deep-rooted causes that connect society, economic and environment (8, 33, 38).

In light of escalating climate risks and shifting political economies of aid and governance, the terrain in which SDP NGOs operate is rapidly changing. This paper explores responses of SDP actors located in a region where climate change has had devastating impacts on national development. The paper critically discusses not only the opportunities for sport-based environmental action, but also the institutional, political, and cultural constraints shaping the emerging frontier. This contribution analyses the responses of SDP actors in Zambia to climate variability and change, with particular focus on recent experiences related to the El Niño-induced drought. To do so, the authors draw upon theories of strategies of choice and environmental structure (39).

### Environmental structure and theories of strategic choice

Giulianotti et al. have raised concerns regarding the limited visibility of strategic choices related to environmental issues within SDP initiatives. This underscores the significance for SDP decision-makers and designers of social intervention programmes to adopt a more deliberate and strategic approach to addressing environmental concerns. Drawing on John Child's theory of strategic choice (40), this paper investigates the fundamental factors contributing to the limited focus on environmental programming within Zambia's SDP sector. Employing the organisational studies framework and specifically addressing the dichotomy between environmental determinism and voluntarism (39), it analyses both the constraints encountered by SDP actors in Zambia and the alternative strategies they have implemented.

Whittington advanced John Child influential article by dispelling the dichotomy, “between *determinism* and *voluntarism—*the former denying, the latter affirming strategic choice” (39), ultimately presenting an alternative realist perspective. In organisational theory, the external environment in which an organisation operates imposes forces beyond the organisation's control. These constraints are understood to limit strategic choices, thereby allowing environmental conditions to influence outcomes. This deterministic orientation negates the significance of individual agency. Conversely, the voluntarist perspective stresses the ability of actors to exercise autonomy in their motivations for action, thereby underscoring the importance of agency in strategic decision-making.

In relation to the Zambian SDP sector, its environment is defined by broader structural conditions that perpetuate the challenges addressed by local development NGOs and the state. These include situational factors affecting target groups, which often act as external constraints or influence how these groups respond to various challenges. Such pre-existing external factors can undermine the capacity of actors, limiting their available choices and shaping the strategies they implement. Hrebiniak and Joyce posit that “at issue is a view of adaptations as a process of reflecting choice and selection vs. one in which it is necessary reaction to peremptory environment forces.” (41).

This paper aims to focus on the aforementioned dichotomy to elucidate the options available to SDP actors concerning environmental stewardship. Although additional conceptual frameworks are available in the strategic choice literature, the authors consider this dichotomy sufficient for analysing why anticipated changes in climate-related behaviours may face situational or structural constraints. It is worth considering whether these very constraints have contributed to the limited emphasis placed on environmental issues.

Beyond presenting a straightforward dichotomy, the authors also examine a realist approach articulated by Whittington based on Roy Bhaskar's work (42), which recognises both structure and agency as integral to understanding human action, specifically in the context of environmental stewardship within the SDP sector. This realist perspective emphasises the significance of pre-existing structures in shaping the exercise of human agency, thereby informing SDP practices and strategic decisions related to environmental concerns.

Drawing on Bhaskar's critical realism, Whittington contends that the “ontological gap between structure and action preserves the possibility of agency.” (39) Echoing Giulianotti’ s use of the socio-ecological approach for examining and advocating for SDP engagement in environmental issues (33), this study uses a realist perspective to show why external structures and environments are critical. Bhaskar's critical realism aids the study to understand how local contexts (i.e., local or national structures) influence SDP actions towards environmental issues (42). Whittington's strategic choice framework equally acknowledges how situated actors operate in cognisant of such structured contexts (39). Therefore, using a realist lens, this study examines how agency and structure interact through SDP actions. It focuses on how actors attempt to adapt to, resist, or transform their context amid the harsh local realities of climate change.

## Methods

To advance the debate initiated by Giulianotti and others concerning the marginalisation of environmental matters within SDP NGOs, this study aimed to investigate and establish the forms of environmental stewardship emerging from the impact of the El Niño drought on SDP operations in Zambia. Previous literature established that the Zambian SDP sector lacked a distinct environmental focus, treating ecological concerns as secondary. Therefore, Giulianotti and others called for future research to consider the political and economic tensions shaping institutional responses to the threat of climate change. To address this gap, this study employed a two-phased mixed methods approach (43) to advance the climate change and SDP sector debate in Zambia. The methods consisted of a survey (Phase 1) and semi-structured interviews (Phase 2).

Phase 1—The rationale for selecting a survey was to gauge the negative impacts of climate change on SDP sector practices and how the sector is responding. The survey tool was specifically designed to capture and provide lived experiences of SDP actors, of the effects of the severe droughts and what practical climate adaptation measures were adopted by the participating organisations. A participant information sheet inviting all SDP organisations that were involved in a local network of civil society actors collaborating on environmental matters was distributed via a localised sport-based social media platform and the National Sport Council of Zambia (NSCZ). The NSCZ distributed the questionnaire to organisations in the civil society sector which were actively working with the council. The researchers directly contacted and invited SDP actors receiving Norwegian Church Aid funding for environmental stewardship initiatives, to respond voluntarily to the survey. Targeting this specific network was theoretically vital, as persistent economic tensions severely constrain environmental programming for both the general SDP sector and NSCZ federations in Zambia. Only ten (10) organisations completed the voluntary questionnaire: 6 of the 10 were SDP NGOs, two (3) were national sports federations, and two (3) were state-sponsored agencies for sport. To align with the focus of the study of extending the debate initiated by Giulianotti and others, the results focus exclusively on the data from the six SDP NGOs.

Phase 2: The quantitative results from Phase 1 informed the purposive sampling (44) strategy for Phase 2. This helped the study to transition from a broad mapping of participating SDP organisations’ responses to more in-depth, contextual understanding of each organisation's climate adaptation response. This involved isolation of cases that demonstrated advanced programming of their environmental stewardship initiatives as a response to the El Nino drought. Two organisations were purposively selected based on their questionnaire responses, which highlighted a concentrated, explicit focus on climate change adaptation and activism. Limiting Phase 2 to only two specific cases allowed for in-depth understanding of organisations that were actively spearheading climate activism within the local SDP network, offering valuable empirical insights into how structural and economic tensions are navigated in practice.

Purposive sampling (44) of two organisations resulted in seeking permission of their leaders knowledgeable (43) about the specific organisational focus on environmental issues, and strategic responses to the local climate-related challenges. Two key informant semi-structured interviews (43) were conducted in English via Microsoft (MS) Teams with each case study organisation, leveraging the researcher's familiarity with the region to facilitate open dialogue. The online interviews, lasting over an hour each, used a flexible, story-telling approach and covered both current issues and historical challenges. Additional written exchanges clarified points after initial data analysis, particularly in interpreting local terminologies which were verified through member-checking against automated transcripts.

The updated and verified MS auto-generated transcripts were analysed employing Braun and Clarke's reflexive thematic analysis (RTA) technique (45, 46, 52). These authors later posit that RTA focuses on “the researcher's reflective and thoughtful engagement with their data and their reflexive and thoughtful engagement with the analytic process”. Two researchers (first and third author) worked on the data independently which included re-listening to the recorded interviews and ensuring that all transcripts were accurate. The locally-based researcher (third author) provided their revised transcripts which were then reviewed by the first author to match their revisions. Thereafter, both researchers discussed the coding of the transcripts insisting on a flexible and organic approach but collaborative. The first author used their experience in qualitative data analysis to provide guidance which focused on using interview items to deductively and inductively interpret the interviewees’ responses. The identification of codes included recognition of climate change impacts, acknowledgement of associated challenges, expressions of agency, environmental determinism, and strategic choice which depicted structure and agency relation, alongside analysis grounded in the data's inherent meaning.

While coders produced differing codes, the first author provided guidance to reconcile keyword groupings. This process identified data patterns that formed the basis for developing themes. The first author's positionality as a Zambian native and SDP lead researcher was carefully considered. While the first author had no prior formal engagement with both the two key interviewees as well as their representative organisations, this author possesses extensive research experience with two SDP NGOs among the questionnaire respondents. To address aspects of power dynamics between interviewee and researcher, the researcher practiced ongoing reflection to ensure that prior experience within the local SDP sector did not unduly influence the interpretation of the data. The generated themes were further reviewed by the second author aiming to identify consensus from both coders. This involved checking for accuracy of interpretation of the quotations used in the draft manuscript, Ethical approval for this study was secured from the first author's relevant institutional ethics review board, as part of a wider activism and SDP project led by the first author. The authors note that while the survey captured the prominent SDP actors within the sector, they acknowledge that the qualitative findings are limited by a small sample size, consisting of only two semi-structured interviews with one senior executive from each of the selected organisation.

## Findings and discussion

This section presents and discusses the findings based on the questionnaire data seeking to identify active responses to the impact of the drought upon SDP sector operations and semi-structured data from two purposively selected organisations. The section presents the findings using the following themes: Environmental issues and SDP climate vulnerabilities; increased SDP participation in environmental stewardship; SDP voluntarism and environmental issues; environmental determinism weakening strategic choice; and lastly, a realist model evident within SDP actors.

### Environmental issues and SDP climate vulnerabilities

All the six SDP NGOs reported that climate change was affecting community sport in direct and practical ways. Specifically, half (3 of 6) of the responding organisations reported a moderate decrease in participation, two reported a slight decrease, and one reported a significant decrease. This indicates that all SDP organisations experienced some level of participation decline. The decline in participation was further defined in terms of participant experiences regarding the quality of sporting activities offered by SDP NGOs. For example, four of the respondents indicated that drought had moderately affected overall enjoyment and quality, while two organisation representatives stated the effect was slight. This result showed that the El Niño drought was not only limiting access to community sport but also impacting the quality of experience within SDP sector activities.

However, it is important to highlight that the physical spaces as sporting activity venues used by SDP organisations are also used by schools; in some instances, venues were shared by both schools and SDP NGOs. Hence, climate vulnerabilities continue to negatively impact the quality of experience of both the SDP sector and school sport provision. Such environmental issues, resulting in drought-induced negative impacts, have forced SDP NGOs to alter their operations by incorporating coping mechanisms to withstand adverse effects. For example, two organisations indicated enforcing changes in playing conditions by making time adjustments, such as shortening events; two organisations reported introducing climate education or awareness activities, and two organisations also referred to tree planting or other environmental actions. Less frequently mentioned responses included emergency planning or backup power measures and playing surface adaptation where only one organisation was reflected in each of those practices.

Zambia's El Niño drought has resulted in water access disruptions emerging as a key SDP operational issue. A majority of respondents, four of the six organisations reported significant challenges in securing sufficient and good-quality water for participant (athlete) hydration and recovery, while only two organisations reported minor challenges. As will be demonstrated later, some SDP NGOs have responded to drought and load shedding by adopting alternative energies, some of which are renewable or green energies, while others are non-renewable. For example, four SDP organisations had adopted solar panels, gas stoves, rechargeable devices, and fuel-powered generators.

The data from the questionnaire responses indicated that the surveyed SDP NGOs were responding to environmental issues. However, the data also indicated that these responses mainly consisted of low-cost and adaptive coping measures as opposed to fully resourced climate resilience planning. The aspect of cost as a resource implication will be discussed later in relation to soft and hard adaptation climate change interventions.

Our findings indicate that in recent years, local SDP actors have become increasingly aware of the severe ramifications on sport programming posed by continued drought and erratic rainfall patterns. These environmental challenges have had a direct impact on the sector's fundamental activities, affecting their core operations from the quality of sports fields to overall levels of engagement and participation. The disruption caused by these climatic changes has not only hindered effective programme delivery but also beginning to impose substantial constraints on individual and community engagement in SDP activities:

The impact of climate change on community sport in our area has been significant, particularly due to recurring drought conditions and the resulting infrastructure strain such as load shedding and water scarcity.

(SDP Climate Change Survey Respondent)

… the playing fields [are] damaged due to heavy rains which has affected the quality of playing surfaces for the participants. The drought has also affected the water table for a number of communities which has led to the negative participation in sports activities as most of the young people cover long distances in search of drinking water.

(SDP Climate Change Survey Respondent)

The El Niño-induced drought in Zambia has significantly disrupted daily routines, influenced attention and fostered local interest regarding environmental matters. Within the SDP sector, both excessive rainfall and prolonged drought conditions were acknowledged by survey respondents as having an impact on the quality of sports fields and suitable playing surfaces. The drought impacting Southern Africa has been particularly significant with regard to food shortages and disruptions in hydroelectric power supply, leading to large-scale electric power load-shedding. Consequently, electricity supply to households, businesses, and public facilities has become inconsistent, attributing it to insufficient water levels in reservoirs required for hydroelectric generation.

For the SDP sector, like other NGOs, day to day desk-based activities and online workshops face significant disruptions due to electricity loadshedding:

we [made] the decision last year to invest in some solar panels because we were finding that the impact of load shedding meant that our staff were unable to work and so we had a whole office full of people waiting to do their work. But we were really reliant on the schedules that were often very temporal… We had to fundraise to get some solar panels on our office, but since having them, it has been a lot better and we can be less reliant on ZESCO [Zambia Electricity Supply Corporation, a state-owned corporation] and power which has enabled us to have more consistent working patterns throughout the week.

(SDP Interviewee 1)

Additionally, the scarcity of water supply due to electricity loadshedding has resulted in SDP NGOs to shorten their sports programmes, directly affecting youth engagement in sport and physical activity. In response, several local SDP NGOs have begun integrating climate education into their initiatives, helping participants understand the negative impacts of environmental issues on their well-being. This study's findings also indicate a possible threat to exacerbating gender inequalities and participation of low-income groups, normally the key target groups for SDP programmes. Those in deprived neighbourhoods are particularly vulnerable to frequent power outages. For instance, a gender-focused organisation reported that the prolonged drought threatens to undermine progress made toward gender equality in sports participation. This respondent noted that corporate sponsors, who previously supported women's sports initiatives, are now withholding funding support due to economic challenges linked to recurrent electricity load-shedding.

…this has negatively impacted the growth of women sport in both economic and social terms. The association depends on corporate sponsorship and most of companies have not performed to their capacity due to power deficit. Further athletes have been affected negativity due to closure of some facilities due to lack of running water as a result of hydroelectricity.

(Climate Change Survey Respondent)

Other SDP sector initiatives such as skill development in ICT for school-based out-reach programmes have equally been affected by the load-shedding of power. SDP organisations which have set-up support structures to enhance the educational achievement and attainment in resource-poor community schools also expressed their disappointment regarding threats to deliver their skills development focused goals due to electricity load-shedding disrupting their ICT activities:

…because of load-shedding, there's often no power in the day, so the ICT class that they normally have is now taught offline and so they might be going through an exercise book to learn about the functions of Microsoft words, things like that… we've run after-school support clubs for exams grade students to help them improve, you know, mathematics, science, ICT support.

(SDP Interviewee 1)

The daily occurrence of electricity load-shedding has become a defining characteristic in communities across Southern Africa. This persistent disruption has had considerable consequences for both social and economic development throughout the region. SDP organisations and their stakeholders have been compelled to reconsider their priorities considering these challenges. Increasingly, there is a movement towards adopting new greener approaches, recognising and actively responding to environmental concerns in their strategic planning of SDP activities. This shift is underpinned by a growing awareness of the essential role that environmental issues play in ensuring the long-term sustainability and success of SDP interventions. The section that follows demonstrates that the recognition of these challenges has led SDP actors to implement targeted strategies that attempt to mitigate the drought-induced challenges faced by their target communities.

### Increasing participation of local SDP in environmental stewardship

In response to environmental concerns, SDP organisations are focused on equipping their staff with relevant knowledge and skills by accessing training from organisations that possess greater expertise capable of supporting SDP climate change related activities. Through these collaborations, SDP staff are potentially better prepared to develop resilient and responsive approaches that reflect the realities of climate change, thereby enhancing chances of the effectiveness of their interventions within affected communities. This was noted when respondents highlighted collaborative initiatives with global organisations operating in Zambia:

We are implementing various measures such as capacity building of staff on climate change, understanding what it is all about, its impact, strategies to address it. This is being done through webinars, summits, enrolling in online trainings. For example, we [NGO staff] are currently attending the online training programme for sports NGOs on climate action by UNITAR.

We have increased our resource mobilisation strategies through grant calls that focus on climate action projects including research grant opportunities to enable us to understand the real impact of climate change in sport in Zambia.

We are [also] sensitizing the community on climate change and SDGs

(SDP Climate Change Survey Respondents)

SDP NGOs are starting to increasingly participate in environmental initiatives at both broad and local levels. Examples include capacity-building interactions with the United Nations Institute for Training and Research (UNITAR) and the local Disaster Management and Mitigation Unit (DMMU) which falls under the Vice President's Office in Zambia. Through the UNITAR relationship, the SDP sector staff, including mainstream NGO workers in Zambia, have access to specialised capacity-building activities. By focusing on equipping staff with relevant knowledge, SDP organisations aim to strengthen their ability to respond to local environmental challenges within the context of SDP and beyond. This may help support securing of adequate capacities necessary to shape the agency of SDP to respond to external constraints (41). The section that follows will show how two SDP organisations demonstrate strategic choice pertaining to environmental issues.

### SDP, voluntarism and environmental issues

This section reports on the actions and omissions of two organisations—Organisation A and Organisation B. Both organisations employ sport as a strategic tool for development, acknowledging the pivotal function of sport in facilitating social mobilisation. Their methodology is founded on the principle that delivering knowledge to foster behavioural change within a sport-oriented setting serves as a practical and effective alternative to traditional approaches. Interview data is drawn upon in this section to illustrate the strategic decisions faced by these SDP actors aimed at demonstrating local agency in environmental issues.

This section tabulates the two organisations’ responses using the frameworks of determinism and voluntarism to analyse and discuss their organisational strategic choices. Determinism serves to elucidate the external constraints faced by organisations, while voluntarism pertains to the exercise of local agency through intentional strategic decision-making (39). Through the application of these concepts, the section attempts to offer a nuanced understanding of how SDP actors balance external restrictions with their own agency in the context of environmental initiatives.

Both organisations (Tables 1, 2) exemplify human agency, as their actions are deliberately chosen in response to environmental circumstances. Strategic decision-making is reflected in their practice of knowledge sharing, which serves to increase awareness and educate their communities about environmental concerns. The voluntaristic initiatives undertaken by both organisations are designed to empower individuals and communities. In the case of Organisation A, this is evident through a self-help approach (47) focused on enabling communities to identify solutions using locally available resources, an essential component of their agency. The prioritisation of local agency over reliance on external resources (48) continues to face environmental limitations, which will be examined further in the context of determinism.

**Table 1:** Voluntarism—organisation A's capacity for independently designed environmental initiatives in accordance with national and local climate adaptations.

Organisation A		
Strategic choice	Description of strategic actions	Voices of agency
Raising awareness of climate issues	Utilising communal gatherings to enhance awareness Adopting a *self-help approach* to empower communities and individuals to recognise their intrinsic potential to address their circumstances by leveraging locally available resources	*“Our role is to raise awareness and make people realise theirinherent potentials.*” “*To do something about their situation or situations, using locally available resources.*”“*We are using the selfhelpapproach yes and in that self-help approach, some of the tenetsor the bedrock of that self-help approach is that our role is not todecide what development people want.*”
Climate smart economic empowerment programme	Leveraging Organisational Capacity to Advance Climate-Smart Initiatives *Drip Irrigation Equipment Initiatives*: Facilitates precision agriculture as part of strategic interventions. *Climate Change Mitigation Programming*: Integrates climate change considerations into all programs as a cross-cutting priority. *Sports Clubs Tree Planting*: Promotes tree planting and active participation in climate initiatives among sports clubs and local communities.	“*So, we supported them with the drip irrigation equipment. You know, so that they exercise precision farming*.” “*Climate change mitigation is more or less a cross-cutting matter.*” “*So, we're encouraging the sports clubs to, for example plant trees around their sports facilities, but not only around the sports facilities but also encourage the communities and plant trees and engage in climate smarter activities just to mitigate the impact.*”

**Table 2:** Voluntarism—organisation B's capacity for independently designed environmental initiatives in accordance with national and local climate adaptations.

Organisation B		
Strategic choice	Description of strategic actions	Voices of agency
Climate change education	*Climate Change Education*: The organisation incorporates climate change awareness into literacy initiatives and reading clubs, as well as site visits and hands-on activities such as recycling and waste collection. These efforts are designed to equip young people with knowledge and capacity to engage meaningfully with environmental challenges. *Agency and Action Empowerment*: Climate-related agency and proactive engagement are fostered through sport-based curricula implemented in community schools.	“*Together, we've really kind of built a more integrated curriculum that is actually now being used in school as well as out of school activities.*”“*We actually run the digital skills innovation hub... to improve employment opportunities for young people to make sure that they have the skills needed.*”“*So, we run projects particularly around our literacy and reading clubs… we've integrated elements of climate change awareness into these clubs… And then also doing site visits, we did one where we went to the Water Works in Livingstone to understand [recycling] we went to recycling plant.*”
School meal feeding programme	*School meals* have been integrated into drought impact mitigation initiatives for young people, with increased provision during the El Niño-induced drought to address heightened food shortages in rural communities. *Food Production Schemes*: School Garden projects are supported through the supply of agricultural implements, aiming to alleviate the challenges presented by load-shedding and water scarcity.	“*And what we often see is that schools that have school feeding programmes have a lot higher attendance rates than those schools who don't offer school meals… and so we've worked with a number of rural schools to help them set up school feeding programmes.*” “*The severe droughts that we saw in those years... we did embark on some forms of more emergency food provision... it wasn't about learning or education at that point, the priority was surviving.*” “*And we've just finished the construction of the school garden…with irrigation solar pumps and irrigation pipes and this is now starting to grow some fruits and vegetables used for their school feeding programme.*”

Nonetheless, effective knowledge empowerment necessitates decision-making and the implementation of practical behavioural changes that are consistent with climate change values. Organisation A operates a climate-smart empowerment programme designed to assist communities through key initiatives, including the promotion of drip irrigation technologies and training to support the adoption of precision agriculture. Both Organisation A and Organisation B collaborate closely with community schools, which are self-organised and independently funded as opposed to government schools. Organisation A also emphasised that a conscious choice was made to prioritise climate change mitigation across all activities. These strategic choices seem to be aligned to national climate adaptation plans, those promoted via the country's national adaptation plan. However, Mweemba terms these approaches as soft climate change adaptation measures which are cheaper to implement compared to ones needing huge capital investments. Soft adaptation interventions in this case refer to climate education, training workshops, and contribution to policy discussion, activities that do not require significant financial resources. Conversely, hard adaptation measures require significant technological and infrastructure commitments (49).

### Environmental determinism weakening strategic choice

This study's voluntarist-oriented findings are promising for the SDP sector, which utilises sport to engage target groups in climate education through adaptation initiatives. Nevertheless, the prevailing impact of environmental factors on the livelihoods of these groups underscores the significant role that context plays in shaping human decisions. Both Organisation A and Organisation B emphasised that environmental conditions impose constraints on stewardship efforts at the individual, community, and organisational levels despite the high climate change awareness:

I don't think there's denial of climate change, the awareness is quite high and this awareness is in most of these communities we are coming across *.*.. of course the government of Zambia, have also taken steps, they talk about climate change through radio programmes… (Organisation A Interviewee—Decision-maker)

The interviewees noted structural constraints, including the impacts of drought and resulting climate-induced poverty, as factors that limit the range of local options for implementing effective climate adaptation strategies. These represent environmental or economic determinants that restrict the choices available for climate change mitigation efforts, as communities weigh what is practical:

The other thing that is affecting or countering the climate change mitigation messages is that there's high dependence on charcoal, for example. This is a practical situation. In our area there's high dependence on charcoal.

(Organisation A Interviewee—Decision-maker)

When climate education is tailored to local contexts in Zambia, it often emphasises sustainable farming techniques and the reduction of deforestation. Nevertheless, the economic imperatives faced by the target population frequently take precedence over climate awareness initiatives as noted below:

And then these communities that are closer to the urban areas find market for charcoal so they are forced to go into tree cutting and burn for charcoal because already the rural communities are impoverished. So that's the source of their livelihood.

So, in spite of them understanding and realising the damage they are creating to their environment, they can't help it because they need to economically survive.

(Organisation A Interviewee—Decision-maker)

The interviewees provided further insight into the economic structural constraints affecting both rural and urban communities, specifically highlighting the elevated cost of electricity tariffs. Climate change has not only disrupted power supplies but has also driven up electricity costs, thereby exacerbating socio-spatial inequalities. Economic determinism was apparent in instances where individuals in rural areas supply charcoal to urban residents, as both groups seek solutions to climate-induced poverty. Although these practices are considered environmentally detrimental and pose challenges for effective local climate education, they underscore the limited survival options available at the community level. The scenario being painted below contributes to a historical economic dynamic between rural and urban populations that has had adverse environmental impacts, as both groups are constrained by limited options:

So shortages of electricity and also the increase in electricity tariffs have made use of electricity expensive, even if it's available. It's one thing with the shortages, it's another thing that electricity is available, but then affordability becomes a factor*.*..

But then among the alternative energies like the greener energy that we are encouraging. It's not capable also to supply energy for cooking for example, you can have the solar system installed in the home, but if you're going to go into cooking, you really need to invest heavily. So that creates demand for charcoal.

(Organisation A Interviewee—Decision-maker)

In these instances, environmental factors play a predominant role in shaping behaviours by influencing strategic decisions aimed at supporting the livelihoods of target audiences. Although economic, environmental, and spatial determinants present complex challenges for SDP actors, the following section briefly discusses how these actors acknowledge their own capacity for agency within existing structures to facilitate such agency.

### A realist model to climate impact mitigation

The actions of both Organisation A and Organisation B demonstrate that these actors have considered the existing social structures as potential avenues endowed with possibilities to not only support their environmental stewardship interventions but also to implement climate adaptation activities. This was observed when actors expressed how organisational capabilities were aligned to external opportunities for climate adaptation. This was based on a recognition of external resources, structures capable of supporting the exercising of agency to widen the strategic choice options for advancing organisational goals by adapting to the context. Each organisation seemed to demonstrate that existing structures enable actions, advancing agency in relation to tackling the implications of the drought upon their operations. This is in line with Bhaskar's realist orientation as both organisations establish their agency by acknowledging structures capable of improving the livelihood of their target audiences.

For example, Organisation A employed a realist orientation when the organisation leveraged and undertook the leadership of activating sub-national structures under the Disaster Management and Mitigation Unit's (DMMU). These structures form part of the national responses to environmental matters. As a DMMU government structure in rural areas, Organisation A used the structural to exercise communal agency:

The DMMU has these structures already, but they're not existent on the ground because they're not reaching out to them…Government was not available because of the remoteness of these areas. There was no technical support, no proper training, so we had to establish these disaster management committees in the villages, so we called the DMMU to train them and come up with early any warning traditional methods and we also trained them in post-harvest management as well.

Funder and Mweemba (26) equally reveal the same that decentralised state structures are rarely visited leaving the task for implementation to interface bureaucrats. In this case Organisation A assumed the responsibility of reviving an existing policy instrument to establish a decentralised structure to expand its strategic choices. By using this local structure, leveraging it to expand strategic choice, Organisation A was able to integrate into national adaptation initiatives. Child asserts that ability to manipulate the environmental features, reviving established structures, is a demonstration of choice of relevant options for organisational performance as a response to environmental matters.

On the other hand, Organisation B demonstrated its realist orientation by collaborating with existing structures as a precondition to their agency to support their target audience develop IT skills or competences. To overcome the limitation brought about by power outages, collaborative working with partners from the financial sector who provided facilities free of electricity power interruptions, enabling consistent access to online computing education. This echoes other studies that have used organisational theory to highlight that aligning to the environment and structural resources supports organisations to achieve optimal performance

A majority of private sector entities have adapted to the load-shedding by investing in greener energy sources such as solar electricity equipment. Agency as demonstrated by Organisation B depicts the expanding of their strategic choice through what Pettigrew (50) terms the use of capitalist structures to enable agency. Similar to Organisation A, this SDP actor widened its strategic choice of serving young people by identifying resources to mitigate the environmental constraints:

…we have been working with [name of financial institution] and we actually run the digital skills innovation hub that they have above their [build] in [name of town]. It's a brilliant space, some really good co working areas. The use of presentation facilities, access to computers and high-speed Internet. And we're working with them to improve employment opportunities for young people to make sure that they have the skills needed.

Both organisations have adjusted to their environment, identifying and leveraging the existing structures as a precondition to their agency. Doing so, has widened their organisational strategic choices through effective mobilisation of structural resources (50), unlike being limited by environmental determinants.

## Conclusion

The aim of this paper was to contribute to the debate regarding the marginalisation of environmental issues by the global SDP sector. This article has highlighted some emerging tendencies among some SDP actors in Zambia, moving environmental matters from the peripherals to part of their SDP activities. The paper considered how local SDP actors have awakened to the call for environmental stewardship via the devastating impact of the El Nino drought in Zambia. It is evident that having been negatively impacted just like the rest of the Zambian society, actors within the SDP sector have had to respond by considering resilient approaches to sustaining their operations.

The premise of the argument regarding SDP and environmental matters by Giulianotti and other was based on questioning the sector's marginalisation of environmental issues despite such matters being key to the SDGs. Giuliannotti further called for a socio-ecological approach to be adopted in attempts to greening SDP, highlighting environmental protection as of the four pillars of the approach. The other pillars being social equality, democracy and social justice.

Using organisational theories of strategic choice, the paper has highlighted the nature of organisational agency and decision-making to engage in environmental concerns. The paper's findings show that environmental stewardship has become an operational necessity, particularly in the face of experienced negative impacts of the drought in Zambia. However, SDP actor decisions to embark upon those courses of strategic action are not free from environment, space and economic constraints.

Some local SDP actors have demonstrated significant voluntarism in their efforts to by-pass inconsistent power grids by fostering relationship with organisations in different sectors which have resources. At the same time, the agency of SDP actors encounters environmental determinism which weakens strategic choice. At individual level, SDP actors aim to alleviate the impact of poverty among their target audiences, to close the gap on socioeconomic equalities. However, this study reveals that immediate economic imperatives frequently overside climate awareness, as survival in strategic choice only leaves participants one environmental detriment option, charcoal production and use of charcoal as fuel.

The paper presented a realist model for operational resilience among SDP actors facing climate challenges. The findings suggest that leveraging of existing social and capitalist structures, supported by policy instruments widens SDP actors’ strategic choice to demonstrate climate reliance. While SDP NGOs and CBOs increasingly use sport to achieve non-sporting outcomes, their efforts tend to lack formal integration into national climate adaptation strategies. SDP scholars argue that while the physical environment plays a key role for delivering SDP activities, environmental matters tend to be overlooked in SDP programming. This neglect highlights the need for policymakers to protect physical spaces, and ensure equitable access to facilities and resources. Such actions, would support the gradual recognition of the potential power of sport as a development tool beyond social and health-related outcomes (4). In the case of Zambia, ensuring the access of sport to constituency development funds (CDF) has been echoed by local SDP actors (51). CDFs are annual financial allocations distributed by the central government to members of parliament for projects approved within their constituencies. SDP actors can play a role as climate adaptation partners as demonstrated through the two organisations in this study.

In conclusion, while some SDP sector actors in this study has demonstrated some remarkable resilience and adaptive capacities, the decision to embark upon strategic environmental action remains heavily constrained by broader environment and socio-economic contexts. Ensuring that the progress noted, of active environmental programming, is sustainable will require not only local agency but also systemic support to realist approaches to tackling environment and economic constraints that currently pit survival against stewardship. This brings us back to the acknowledgement that SDP has embarked on tackling social issues that are complex with deep-rooted causes. SDP can contribute to solutions but only through sustainable interdisciplinary or integrated approaches.

## Data Availability

The original contributions presented in the study are included in the article/Supplementary Material, further inquiries can be directed to the corresponding author/s.
