# Supplementary material for: Sport for development sector and climate change adaptation in Zambia—theories of strategic choice
Source: Front Sports Act Living. 2026 Jul 1;8:1756982. doi: 10.3389/fspor.2026.1756982 (PMC13383100; doi:10.3389/fspor.2026.1756982)
Supplement: Supplementary file 1 [file Datasheet1.pdf]

Questionnaire Survey: Impacts of Climate Hazards and Power Outages on Community Sport Organisations in Zambia

This survey aims to understand how community sport organisations in Zambia have been affected by climate hazards (droughts and floods) and power outages. Your experiences will help us to better understand the challenges faced by community sport and inform future support initiatives. All responses will be kept confidential.

Instructions: Please tick the box that best reflects your experience or opinion. For open-ended questions, please provide as much detail as possible.

Section A: Respondent Information

1. **Select the nature of your organisation?** (Tick one)
   - [ ] State sponsored sport organisation
   - [ ] Non-governmental sport organisation
   - [ ] Professional Sport Association
   - [ ] Sport Governing Body
   - [ ] Other (Please specify: __________)
2. **What type of sport(s) are you primarily involved in?** (Please specify: __________)
3. **State the location of your organisation** (Province/City/District) ___________________
4. **Number of years your organisation has been active in community sport:**
   - [ ] Less than 1 year
   - [ ] 1-5 years
   - [ ] 6-10 years
   - [ ] More than 10 years

Section B: Impact of Drought on Community Sport

4. **Over the past 5 years, how has drought impacted the availability of suitable playing fields/surfaces for community sport in your area?**
   - [ ] No impact
   - [ ] Minimal impact
   - [ ] Moderate impact (e.g., occasional closures, reduced quality)
   - [ ] Significant impact (e.g., frequent closures, unusable fields)
   - [ ] Severe impact (e.g., complete lack of suitable fields)
5. **Has drought led to any changes in the frequency or duration of community sport activities (e.g., training sessions, matches)?**
   - [ ] No change
   - [ ] Reduced frequency
   - [ ] Shorter duration
   - [ ] Complete cancellation of some activities
   - [ ] Other (Please specify: __________)
   - [ ] Complete lack of water
   - [ ] Not applicable (no facilities)
6. **Has access to water for hygiene and sanitation at community sports facilities been affected by climate change and/or drought?**

- [ ] No impact
- [ ] Minor inconvenience
- [ ] Significant limitations
- [ ] Complete lack of water
- [ ] Not applicable (no facilities)

7. **Has climate change, including drought, impacted the participation levels in community sport in your area?**
   - [ ] No impact
   - [ ] Slight decrease
   - [ ] Moderate decrease
   - [ ] Significant decrease
   - [ ] Increase (Please explain why: __________)
8. **Have there been any climate-related events (e.g., heatwaves, floods, water scarcity, or cholera) that have led to health or safety concerns for community sport participants?**
   - [ ] No
   - [ ] Minor concerns
   - [ ] Moderate concerns
   - [ ] Significant concerns
9. **Has drought impacted the participation levels in community sport in your area?**
   - [ ] No impact
   - [ ] Slight decrease
   - [ ] Moderate decrease
   - [ ] Significant decrease
   - [ ] Increase (Please explain why: __________)

10. **Have there been any conflicts or tensions caused by drought or load shedding such as demands on the time of programme participants by key stakeholders such as parents/guardians or schools which has resulted in limited access to the participants availability for community sport?**
    - [ ] No
    - [ ] Minor tensions
    - [ ] Moderate conflicts
    - [ ] Significant conflicts
    - [ ] Other (Please specify: __________)

11. **To what extent has drought affected the overall enjoyment and quality of community sport experiences?**
    - [ ] Not at all

- [ ] Slightly
- [ ] Moderately
- [ ] Significantly

12. **What coping mechanisms, if any, have community sports organisations in your area adopted to deal with the impacts of drought?** (Open-ended)

---

11. **Has drought led to any changes in the scheduling or format of community sport provision by your organisation?**
    - [ ] No change
    - [ ] Rescheduling of events
    - [ ] Shortening of events
    - [ ] Cancellation of events
    - [ ] Changes in playing conditions (e.g., dry pitches)
    - [ ] Other (Please specify: __________)

12. **Has access to sufficient and quality water for athletes' hydration and recovery been a challenge for community sport provision due to drought and subsequent load shedding?**
    - [ ] No
    - [ ] Minor challenges
    - [ ] Significant challenges
    - [ ] Severe challenges

13. **Has drought and load shedding impacted the attendance of fans, or supporters at your sports competitions?**
    - [ ] No impact
    - [ ] Slight decrease
    - [ ] Moderate decrease
    - [ ] Significant decrease
    - [ ] Increase (Please explain why: __________)

14. **What strategies have your community sports organisations implemented as a response to the impact of drought and load shedding of power by ZESCO?** (Open-ended)

Section C: Awareness of Climate initiatives and Sustainable Sport in Drought Emergencies

15. **To what extent do your current community sport programmes by your organisation address the potential impacts of drought on sport?**
    - o [ ] Not at all
    - o [ ] Minimally
    - o [ ] Moderately
    - o [ ] Significantly
    - o [ ] I am unaware of any such initiatives
16. **In your opinion, how important is it for your sport programming to demonstrate awareness of climate change impacts and to explicitly consider planning for drought or flooding emergencies?**
    - o [ ] Not important
    - o [ ] Slightly important
    - o [ ] Moderately important
    - o [ ] Very important
    - o [ ] Extremely important

17. **What specific measures is your organisation currently undertaking to demonstrate responses to drought emergencies and ensure sustainable community sport provision?** (Please provide specific examples)

    ---

20. **Is your community sport organisation currently undertaking any initiatives or practices that contribute to environmental sustainability?** (Tick all that apply)
    - o [ ] Water conservation measures (e.g., rainwater harvesting, efficient irrigation)
    - o [ ] Energy efficiency measures (e.g., solar panels, energy-efficient lighting)
    - o [ ] Waste reduction and recycling programs
    - o [ ] Promotion of sustainable transportation (e.g., encouraging cycling,)
    - o [ ] Sustainable procurement practices (e.g., buying locally, choosing eco-friendly products)
    - o [ ] No, not currently
    - o [ ] Other (Please specify: __________)
21. **What are the main barriers or challenges that prevent community sport organisations in your area from implementing more sustainable practices?** (Tick all that apply)
    - o [ ] Lack of funding
    - o [ ] Lack of knowledge or awareness
    - o [ ] Lack of time or volunteers
    - o [ ] Lack of infrastructure or resources

- [ ] Not seen as a priority
- [ ] Other (Please specify: __________)

22. **What types of support or resources would help community sport organisations in your area to become more environmentally sustainable?** (Open-ended)

---

23. **Elaborate how your organisation has partnered or worked collaboratively with other organisations (donors or government) to demonstrate awareness and responses to mitigate against climate change impacts on community sport.** (Please provide specific examples)

End of survey
